# The Synthesis and Accumulation of Resveratrol Are Associated with Veraison and Abscisic Acid Concentration in Beihong (*Vitis vinifera* × *Vitis amurensis*) Berry Skin

**DOI:** 10.3389/fpls.2016.01605

**Published:** 2016-11-03

**Authors:** Junfang Wang, Shuqin Wang, Guotian Liu, Everard J. Edwards, Wei Duan, Shaohua Li, Lijun Wang

**Affiliations:** ^1^Beijing Key Laboratory of Grape Science and Enology and Key Laboratory of Plant Resources, Institute of Botany, Chinese Academy of SciencesBeijing, China; ^2^Viticulture and Enology Research Center, Institute of Agro-food Science and Technology, Shandong Academy of Agricultural SciencesJinan, China; ^3^College of Life Sciences, University of Chinese Academy of SciencesBeijing, China; ^4^Group of Grapes and Horticulture, CSIRO Agriculture, Glen OsmondSA, Australia

**Keywords:** abscisic acid, veraison, resveratrol, anthocyanin, grape

## Abstract

Resveratrols are polyphenolic secondary metabolites that can benefit human health, and only occur in a few plant families including Vitaceae. It has been reported that abscisic acid (ABA) can induce veraison (the onset of grape berry ripening) and may induce the accumulation of resveratrol in berry skin. However, the relationships between ABA, veraison, the accumulation of anthocyanins and the accumulation of resveratrol in the berry are poorly understood. This study attempted to answer this question through an investigation of the effect of applied ABA and fluridone (a synthetic inhibitor of ABA) on the biosynthesis and accumulation of ABA, anthocyanin, and resveratrol in Beihong (*Vitis vinifera* × *Vitis amurensis*) berry skin. Under natural conditions, resveratrol concentration was very low before 91 DAA (days after anthesis), i.e., 2 weeks after veraison, however, it increased sharply from this point to 126 DAA (maturity). Exogenous ABA applications all resulted in an increase in berry skin ABA and anthocyanin concentration, irrespective of the developmental stage at which the treatment occurred (20 and 10 days pre-veraison, veraison or 7 days post-veraison), thereby advancing veraison. In contrast, resveratrol concentration increased only when ABA was applied at 10 days pre-veraison or at veraison. As a result, the accumulation of resveratrol was associated with veraison in grape berry skin and this accumulation, together with that of anthocyanins, was associated with ABA concentration. The response of resveratrol biosynthesis in the berry skin to manipulation of ABA varied during berry development and was less sensitive to ABA than the response of anthocyanin biosynthesis.

## Introduction

Stilbenes are polyphenolic secondary metabolites occurring in a few plant families including Vitaceae (Jeandet et al., 2002). Grapevine stilbenes have been the subject of many studies due to their function as phytoalexins with a broad spectrum activity (Jeandet et al., 2002; Adrian and Jeandet, 2006). In addition, several members of the stilbene family including resveratrol (3,5,4′-trihydroxystilbene) have been found to have beneficial effects on human health (Aggarwal et al., 2004). Many studies have reported that resveratrol can prevent or slow the progression of a wide variety of illnesses including cancer and cardiovascular diseases (Lee et al., 2012; Csuk et al., 2013).

Interest in resveratrols has been growing since these compounds were found to accumulate in significant amounts, not only in vegetative parts of grape such as stems and leaves, but also in fruits (Wang et al., 2010). In general, resveratrol assigns *trans*-resveratrol (*trans*-Res), *cis*-resveratrol (*cis*-Res), *trans*-piceid (*trans*-Pd) and *cis*-piceid (*cis*-Pd) and the naturally occurring concentrations of these compounds vary between tissues and genotypes (Li et al., 2006; Wang et al., 2013b). Phenological stage also affects tissue concentration, with an increase in *trans*-Res content reported late in the berry ripening phase in *Vitis vinifera* fruit (Moreno et al., 2008). Further, Gatto et al. (2008) reported much higher resveratrol concentrations after veraison (the onset of ripening in the grape berry), the onset of ripening, than at veraison in 78 *V. vinifera* cultivars, although there were large, significant differences among genotypes in the concentration of resveratrols in ripe berries. Therefore, although there is already some evidence for the role of phenological stage, the dynamics of resveratrol accumulation during berry development require further study. In particular, whether, and to what extent, resveratrol synthesis is directly associated with veraison.

Grape berry ripening involves the integration of multiple hormone signals, such as ABA, brassinosteroids (BR), and ethylene, which have all been implicated in this process (Davies et al., 1997; Chervin et al., 2004; Symons et al., 2006; Nicolas et al., 2014). Given that berry resveratrol content also appears to vary with phenological stage (see above) our hypothesis was that resveratrol concentration would be regulated by those same plant hormones and synthesis likely triggered with veraison.

During veraison, the concentration of some of these hormones can change abruptly, in particular, the ABA content of grape berries increases rapidly just before veraison (Coombe, 1992; Antolín et al., 2003; Deluc et al., 2009). Numerous reports have suggested that this increase in ABA may play a major role in controlling several ripening-associated processes in the berry, including coloration, sugar accumulation, and softening (Coombe, 1992; Davies et al., 1997; Giovannoni, 2001; Rodrigo et al., 2003; Yu et al., 2006; Wheeler et al., 2009; Gambetta et al., 2010; Giribaldi et al., 2010; Gagné et al., 2011).

Consequently, the relationship between the resveratrol and ABA in the berry was the focus of our work, the objective of which, was to determine the detailed dynamics of resveratrol concentrations during berry development and maturation, the association between the onset of resveratrol synthesis and veraison, and the relationship, if any, between the accumulation of resveratrol and the concentration of ABA in the grape berry. This was achieved through the application of exogenous ABA and an inhibitor of ABA synthesis at different stages of berry development, then tracking the concentrations of resveratrol and ABA in those fruit through maturity to harvest. In addition the expression of a wide range of genes thought to regulate resveratrol synthesis were monitored.

## Materials and Methods

### Plant Materials and Treatments

Berries of the wine-making cultivar ‘Beihong’ were used in this study. ‘Beihong’ is a cross between ‘Muscat Hamburg’ (*V. vinifera* L.) and wild *V. amurensis* Rupr. generated by the Institute of Botany, Chinese Academy of Sciences, and approved as a new grape cultivar in China in 2008 (Fan et al., 2009). Vines in this study were planted in the spring of 2003 in the experimental vineyard of the Institute of Botany, Beijing, China. The vineyard received local standard management practices including irrigation (kept grapevines from drought stress), fertilization, pruning, and disease control.

The experiments were divided into two parts. Part I assessed soluble solids in berry juice and ABA, anthocyanin and resveratrol accumulation in berry skins throughout berry development. Part II assessed the effect of exogenous ABA on ABA, anthocyanin and resveratrol synthesis and accumulation in berry skin. For Part I, the berries were sampled at different development stages under natural conditions. Clusters, chosen at random, were harvested at 40, 50, 60, and 70 DAA, and at 1 week intervals after veraison (70 DAA) until maturity (126 DAA). The veraison date was determined as the date when 50% of the berries had a visible change color. The maturity date was based on the seed color changing to dark brown without senescence of berry tissue and on previous records. Three replicates per cluster were sampled at each date. After the clusters were harvested, the berries were peeled rapidly, and the skins were frozen in liquid nitrogen. Samples were stored at -80°C until use. For part II, berry clusters at a similar developmental stage were chosen for ABA and fluridone (an inhibitor of ABA synthesis) application. Four treatment stages were at 20, 10 days pre-veraison, version and at 7 days post-veraison. 500 mg L^-1^ (+)-ABA or fluridone solution was used to soak berry clusters for 1 min. All treatments occurred after nightfall. Purified water was used as the control. Samples were collected in the morning (6:00–7:00) at 0, 1, 3, 5, 7, 14, 21, 28 days after treatment and maturity. All treatments had three independent replicates, and each cluster was a replicate (consisting of ∼50 berries) and from different vines. The clusters were taken to the laboratory immediately after being harvested, to take photographs, and washed using running potable water, then washed using deionized water (to remove any residual exogenous ABA or ABA inhibitor solution on the berry surface). Berry skins were collected and stored as described for Part I.

### Abscisic Acid and metabolites quantification

Abscisic acid and its metabolites were quantified according to the method of Speirs et al. (2013). In brief, berry skins were freeze-dried and coursely ground berry skins were ground to a fine powder. A 50–100 mg sample was used for the extraction, to which a deuterated internal standard was added (400 μl, containing D3-7′, 7′, 7′-PA (phaseic acid) and -dihydrophaseic acid, D5-4, 5, 8′, 8′, 8′-ABA-GE, and D6-3′, 5′, 5′, 7′, 7′, 7′-ABA). Phenomenex SPE columns (60 mg mL^-1^: 8B-S100-UAK) were used to provide an initial sample ‘clean-up’ step prior to analysis. The samples were loaded onto the columns, washed with 20% aqueous methanol, and eluted with 90% aqueous methanol. An aliquot of the eluate was dried in a vacuum centrifuge in preparation for analysis.

Abscisic acid concentration was quantified by LC-MS/MS. The dried berry skin extracts were dissolved in aqueous acetonitrile (10% with 0.05% acetic acid) and analyzed by LC-MS/MS (Agilent 6410). Separations were carried out on a Phenomenex C18 column at 40°C, using a gradient from 10% acetonitrile, 90% water and ending at 90% acetonitrile, 10% water. Compounds were identified by retention times and multiple reaction monitoring.

### Resveratrol Quantification

Each sample was extracted for 24 h in methanol and ethyl acetate (1/1, v/v; 1000 mg per 10 ml of organic solvent) at 25°C in the dark (Liu et al., 2013). The suspension was centrifuged at 10000 × *g* for 10 min. The supernatant was separated and the resulting residue was extracted with 3 ml methanol and ethyl acetate (1/1, v/v). The organic solvent phases were pooled and vacuum-dried in a rotary evaporator (N-1001D-WD, EYELA, Tokyo Rikakikai, Japan) at 40°C. The dried samples were re-dissolved in 2 ml pure methanol and stored at -40°C for resveratrol analysis.

Each sample was filtered through a 0.22 μm PTFE membrane filter. Resveratrol was analyzed in a Dionex P680 HPLC system (Dionex Corporation, Sunnyvale, CA, USA) coupled to a Dionex PDA-100 detector. Separation was achieved using a reverse-phase C18 column of Atlantis^®^ T3 (4.6 mm × 250 mm, 5.0 μm particle size, Waters, USA) and a guard column (Atlantis T3, 4.6 mm × 20 mm, 5.0 μm cartridge, Waters, USA) maintained at 30°C with a Dionex TCC-100 thermostat column. The injection volume was 10 μl. Separation was performed at a flow rate of 1.0 ml min^-1^ with the mobile phase consisting of H_2_O (A) and acetonitrile (B). The solvent gradient was performed in the following manner, 0–5 min with 10–17% solvent B; 5–12 min with 17–18% solvent B; 12–22 min with 18–22% solvent B; 22–30 min with 22–33% solvent B; 30–45 min with 33–38% solvent B; 45–50 min with 38–80% solvent B; 50–53 min with 80–10% solvent B; and 53–60 min with 10% solvent B. For fluorimetric detection, the maximum absorption wavelength of two *trans*-isomers (i.e., *trans*-Res, and *trans*-Pd) was 306 nm and of two *cis*-isomers (i.e., *cis*-Res and *cis*-Pd) was 288 nm. Each sample was also scanned from 240 to 600 nm.

*Trans*-resveratrol and *trans*-piceid standards were purchased from Sigma-Aldrich (St. Louis, MO, USA) and the Chinese Standards Research Institute, respectively. The mixed solution of the two *trans*-isomer standards were partly converted to the two *cis*-isomers after UV-C irradiation for 30 min at 6 W m^-2^ and a distance of 15 cm. Conversion coefficients were computed from the two *trans*-isomers; standard curves of the four isomers were generated. However, we only detected *trans*-res, *trans*-pd, and *cis*-pd in the samples. Therefore, the total content of resveratrol was obtained from the sum of these three isomers.

### Anthocyanin Quantification

Anthocyanin relative concentration and composition were determined by HPLC as in Guan et al. (2012). The frozen samples were ground to a fine powder in liquid nitrogen using an A11 Basic Analytical Mill (IKA, Staufen, Germany). One gram of skin powder was extracted twice in 10 mL methanol (2% formic acid v/v) with shaking in darkness for 24 h at 4°C. Extracts were then centrifuged at 12000 × *g* for 15 min. The supernatant was concentrated under vacuum at 39°C using a rotary evaporator (RE-52AA; YaRong, Shanghai, China) until dryness. The dry residue was dissolved in 4 mL deionized water, and then 1 mL was passed through a 0.22 μm Millipore filter for HPLC analysis. Samples were analyzed using an HPLC fitted with a PDA detector (1290 series; Agilent, Palo Alto, CA, USA) with a reversed phase C18 column Kromasil-100 (5 μm particle size, 250 mm × 4.6 mm i.d.; Tracer Analitica, Barcelona, Spain) protected by a C18 Nova Pack guard precolumn (Waters, Milford, MA, USA). The separation used aqueous 2.5% formic acid (solvent A) and acetonitrile containing 2.5% formic acid (solvent B). The gradient was from 10 to 15.8% B for 7 min; from 15.8 to 17.3% B for 12 min; from 17.3 to 20% B for 3 min; from 20 to 22.3% B for 9 min; from 22.3 to 23% B for 9 min; isocratic 23% B for 20 min; and then returned to initial conditions for 4 min at a flow rate of 0.3 ml min^-1^. Injection volumes were 5 μl and the column temperature was set at 30°C. Anthocyanins were detected by UV absorbance at 280 and 520 nm. The identification of anthocyanins was carried out by LC-MS/MS (Guan et al., 2012), supported by spectral analysis and comparison of the elution time and absorbance spectra with a commercial standard of malvidin-3-*O*-glucoside chloride (PhytoLab Gmbh, KG, Dutendorfer, Germany). The concentration of individual anthocyanins was expressed as malvidin-3-*O*-glucoside chloride equivalents calculated from the standard.

### Preparation of Total RNA and cDNA and qRT-PCR Analysis

Total RNA was extracted using a General Plant Total RNA Extraction Kit (Bioteke, Beijing, China). Grape berry cDNA was prepared using the Reverse Transcription Kit (Fast quant RT with gDNase KR106, Tiangen, Beijing, China). Briefly, 2 μl of 5 × DNA Buffer was mixed with 13 μl of total RNA and incubated for 3 min at 42°C. This solution (15 μl) was mixed with 2 μl of 10× Fast RT Buffer, 1 μl RT Enzyme Mix, and 2 μl FQ-RT Primer Mix and incubated for 15 min at 42°C and for 3 min at 95°C to produce cDNA.

The expression levels of *Actin. STS. 3-O-GT* (3-*O*-β-glycosyltransferases), *Myb14. CHS* (chalcone synthase), *UFGT* (UDP-glucose: flavonoid 3-*O*-glucosyltransferase), *MybA1. NCED1*, and *NCED2* were analyzed. The primers (**Supplementary Table S1**) were designed based on previous studies (Wang et al., 2013a; Zheng et al., 2013). The qRT-PCR system consisted of a Rotor-Gene 3000 Amp PCR system (Agilent Technologies); a real-time PCR Kit (Real master mix with SYBR Green FP202, Tiangen, Beijing, China) was used. The amplification of the *actin* rRNA gene was used as an internal control and for data normalization (Reid et al., 2006; Jarošová and Kundu, 2010; Wang et al., 2014).

### Statistical Analyses

Data were analyzed by ANOVA, Student–Newman–Keuls’s multiple range tests (at *P* < 0.05), and paired-sampled *T*-test (at *P* < 0.05 and *P* < 0.01) using the SPSS 13.0 program (SPSS, USA).

## Results

### Soluble Solids, ABA, Anthocyanin and Resveratrol Concentration during Berry Development

**FIGURE 1:**
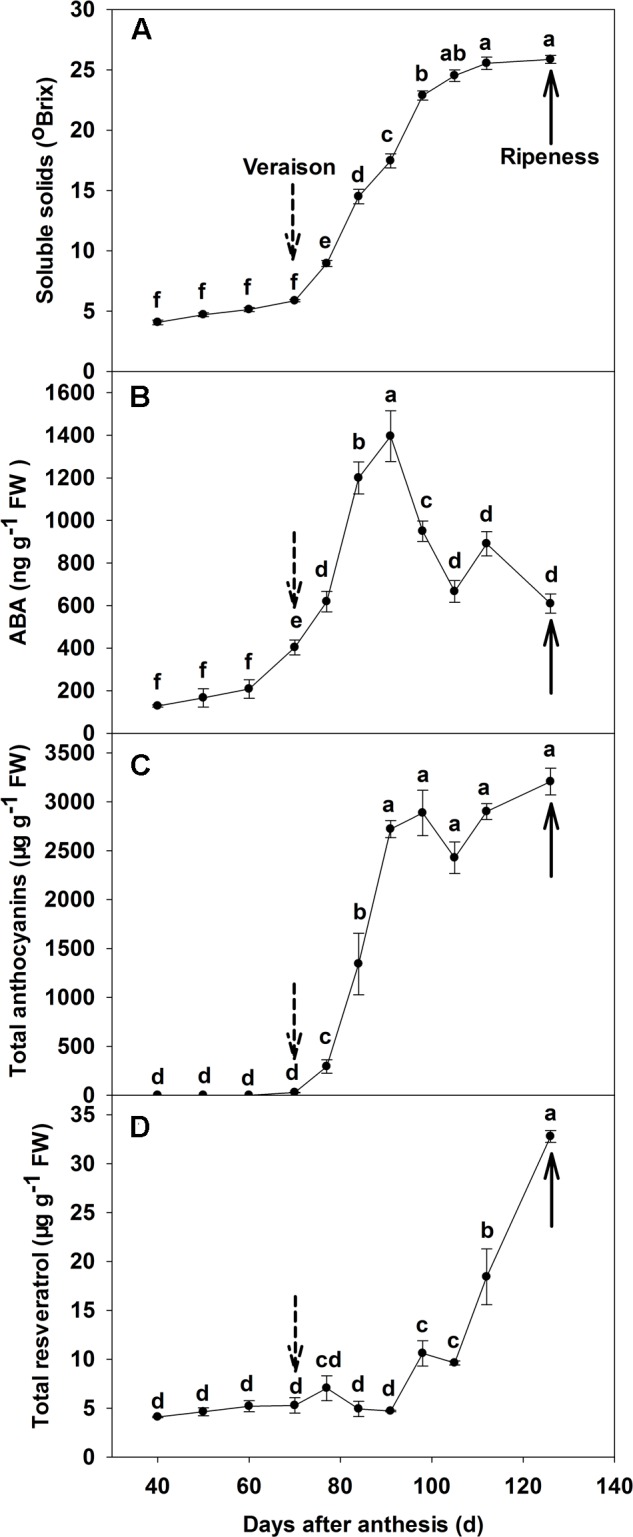
**Dynamic changes of soluble solids** **(A)**, ABA **(B)**, total anthocyanin **(C)** and total resveratrol **(D)** concentrations throughout berry development of ‘Beihong’ (*Vitis vinifera* × *Vitis amurensis*). Data are mean of three replicates and the bar represents standard error (SE). FW, fresh weight; ABA, abscisic acid. Dotted arrow indicates the beginning of veraison, solid arrow indicates the ripeness (completion of the ripening process). Different letters indicate the significant differences (*P* < 0.05) of souble solids, anthocyanin, ABA, or resveratrol concentrations among different development stages.

The total soluble solid of ‘Beihong’ berry increased from 5.8°Brix at veraison (70 DAA), to 25.5°Brix at 112 DAA, with no further increase to maturity at 126 DAA (**Figure 1A**). Free ABA concentration was low prior to veraison (**Figure 1B**), but increased to approximately 400 ng g^-1^ fresh weight (FW) at veraison, and continued to increase to approximately 1400 ng g^-1^ FW at 91 DAA. After this point, ABA concentration decreased to maturity, but remained at a higher level than pre-veraison. The concentration of anthocyanins was very low before veraison, increased rapidly from 10 μg g^-1^ FW at veraison to 3000 μg g^-1^ FW at 98 DAA, and then continued to increase to maturity at a very slow rate, reaching 3470 μg g^-1^ FW (**Figure 1C**). Resveratrol concentration was low until 91 DAA (2 weeks after veraison), ranging from 5 to 8 μg g^-1^ FW, but increased sharply to 35 μg g^-1^ FW at maturity (**Figure 1D**).

### Effect of Exogenous ABA and Its Inhibitor Fluridone on ABA Concentration in Berry Skin at Different Development Stages

**FIGURE 2:**
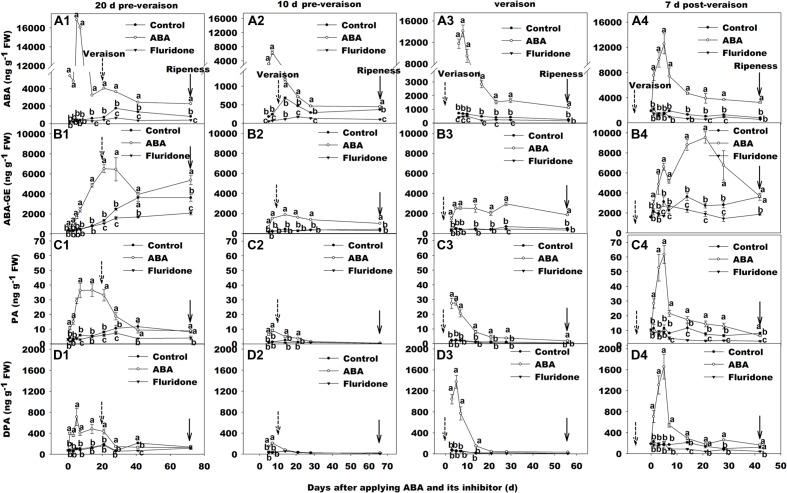
**Dynamic changes of ABA and its metabolism concentration in berry skin of ‘Beihong’ (*V. vinifera* × *V. amurensis*) after subjected to exogenous ABA and its synthetic inhibitor fluridone at 20 days pre-veraison** **(A1, B1, C1, and D1)**, 10 days pre-veraison **(A2, B2, C2, and D2)**, veraison **(A3, B3, C3, and D3)**, and 7 days post-veraison **(A4, B4, C4, and D4)**. Data are mean of three replicates and their SE. FW, fresh weight. ABA, abscisic acid; ABA-GE, ABA-glucose ester; PA, phaseic acid; DPA, dehydro-phaseic acid; Control, deionized water; ABA treatment, (+)-abscisic acid (500 mg L^-1^); Fluridone, an inhibitor of ABA synthesis (500 mg L^-1^). Dotted arrow indicates the beginning of veraison, solid arrow indicates the ripeness (completion of the ripening process). Different letters indicate the significant differences (*P* < 0.05) of ABA, ABA-GE, PA or DPA concentrations among different treatments at each development stage.

We applied exogenous ABA to grape berries at 20, 10 days before veraison, at veraison and 7 days after veraison. The concentration of ABA in berry skin increased in each stage of development (**Figures 2A1–A4**), reaching as high as 16000 ng g^-1^ FW (about 26-fold higher than the control) at 20 days pre-veraison application (**Figure 2A1**). It was notable that in all four treatments, peak ABA concentration was not reached until approximately 5–7 days after ABA was applied. After this time ABA concentration in berry skin declined irrespective of the timing of application, but remained higher than controls for at least 20 days or, in three of the four applications, until maturity. The concentration of ABA-GE was also increased after applying ABA in all four treatments, while the peak time was latter than that of ABA (**Figures 2B1–B4**). However, the change trend of PA (**Figures 2C1–C4**) and dehydro-phaseic acid (DPA; **Figures 2D1–D4**) concentration was very similar. In addition, their peak time was almost accordance to that of ABA concentration.

We also treated grape berries with fluridone at the same developmental stages as the exogenous ABA applications. This resulted in lower berry skin ABA concentration than control in each case, although the timing and duration of the difference varied according to the developmental stage (**Figures 2A1–A4**). When the fluridone treatment occurred at 20 days pre-veraison, where the ABA concentration of controls was very low, there was no significant difference until 28 days after application, coinciding with the increase in ABA associated with veraison, but the difference was maintained until maturity (**Figure 2A1**). The fluridone treatment at 10 days pre-veraison had a much more immediate effect on ABA compared with control, but was applied at a time when ABA in the control was already increasing (**Figure 2A2**). In contrast, when applied at veraison or 7 days post-veraison, the inhibitor had no significant influence on the concentration of ABA (**Figures 2A3,A4**).

**FIGURE 3:**
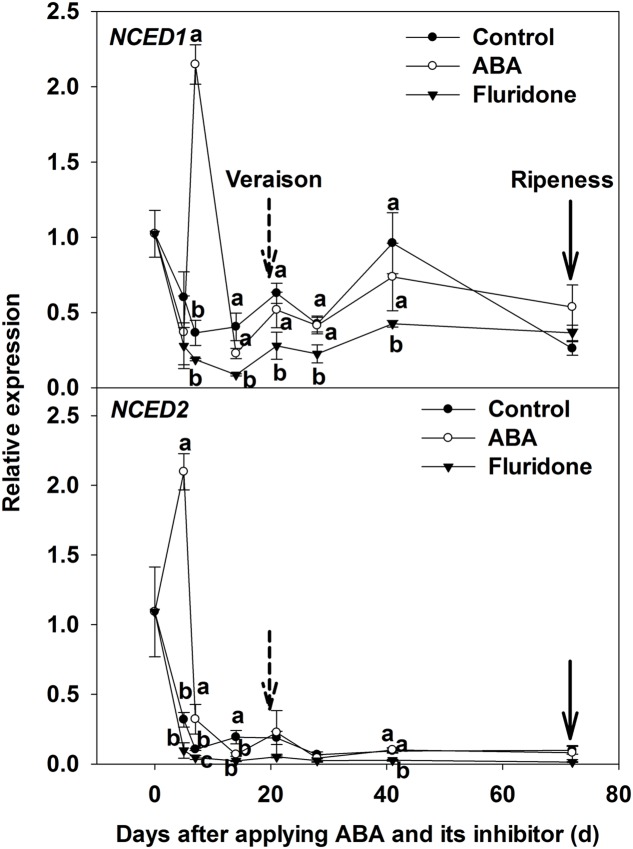
**Expression of genes *NCED1* and *NCED2* related to ABA synthesis in berry skin of ‘Beihong’ (*V. vinifera* × *V. amurensis*) after subjected to ABA and its synthetic inhibitor fluridone at 20 days pre-veraison.** NCED, 9-*cis*-epoxycarotenoid dioxygenase. Control, deionized water; ABA treatment, (+)-abscisic acid (500 mg L^-1^); Fluridone, an inhibitor of ABA synthesis (500 mg L^-1^). Dotted arrow indicates the beginning of veraison, solid arrow indicates the ripeness (completion of the ripening process). Different letters indicate the significant differences (*P* < 0.05) of *NCED1* and *NCED2* expression among different treatments at each development stage.

In addition, we measured the expression of ABA biosynthesis genes (*NCED1* and *NCED2*) in berry skins when exogenous ABA treatments were conducted at 20 days pre-veraison. At 5 days after ABA application, the expression of *NCED2* in the treatment was higher than in the control; while at 7 days after ABA application, the expression of *NCED1* in the treatment was higher than in the control. The expression of both genes then declined and remained at a very low level until maturity (**Figure 3**).

### Effect of exogenous ABA and its inhibitor fluridone on anthocyanin concentration in berry skin during different development stages

**FIGURE 4:**
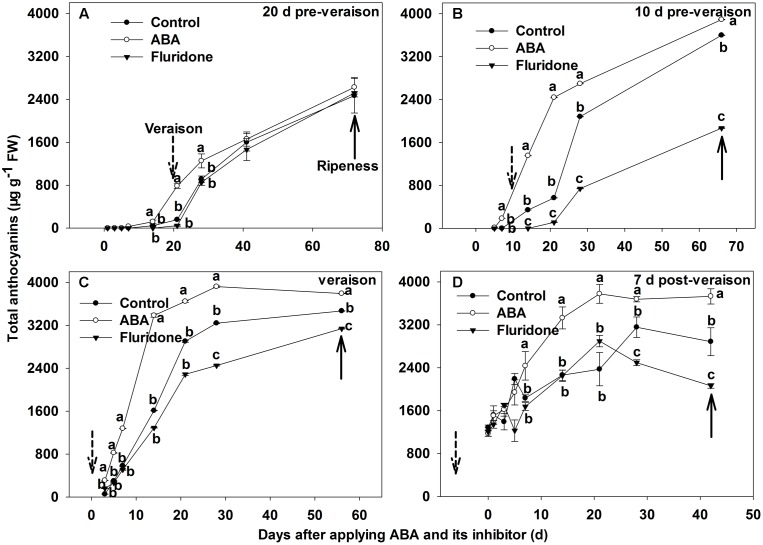
**Dynamic changes of total anthocyanin concentration in berry skin of ‘Beihong’ after applied with ABA and its synthetic inhibitor fluridone at 20** **(A)**, 10 **(B)** days pre-veraison, veraison **(C)**, and 7 days post-veraison **(D)**. Control, deionized water; ABA treatment, (+)-abscisic acid (500 mg L^-1^); Fluridone, an inhibitor of ABA synthesis (500 mg L^-1^). Dotted arrow indicates the beginning of verasion, solid arrow indicates the ripeness (completion of the ripening process). Different letters indicate the significant differences (*P* < 0.05) of total anthocyanin concentration among different treatments at each development stage.

We further investigated the change in total anthocyanins concentration of the berry skins after ABA application. The concentration of total anthocyanins in berry skins exhibited a large increase in each case (**Figure 4**). Following the 20 days pre-veraison application, there was no significant difference in total anthocyanins concentration between the treatment and control from 1 to 14 days after the treatment. From 14 days after the treatment, total anthocyanins concentration in the treated skin increased until maturity. At 21 and 28 days after the treatment, total anthocyanins concentration in the treated skin was higher than in the control (**Figure 4A**). When exogenous ABA applications were conducted at 10 days pre-veraison, veraison and 7 days post-veraison, the skins of ABA-treated berries had significantly higher anthocyanin concentration than the control from 7 days (3 days for the application at version) to maturity after the treatment (**Figures 4B–D**).

Fluridone application at 20 days pre-veraison had no significant effect on anthocyanin concentration (**Figure 4A**). In contrast, fluridone delayed anthocyanin synthesis and accumulation in berry skin after application at 10 days pre-veraison, veraison or 7 days post-veraison (**Figures 4B–D**).

**FIGURE 5:**
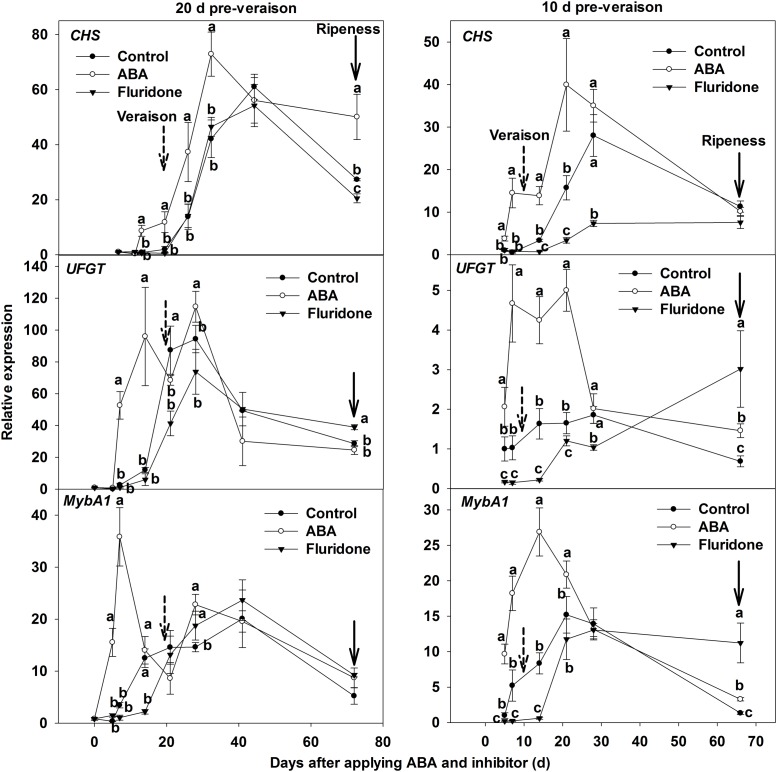
**Expression of genes *CHS. UFGT*, and *MybA1* related to anthocyanin synthesis in berry skin of ‘Beihong’ after applied with ABA and its synthetic inhibitor fluridone at 20 and 10 days pre-veraison.** CHS, chalcone synthase; UFGT, UDP-glucose: flavonoid 3-*O*-glucosyltransferase. Control, deionized water; ABA treatment, (+)-abscisic acid (500 mg L^-1^); Fluridone, an inhibitor of ABA synthesis (500 mg L^-1^). Dotted arrow indicates the beginning of veraison, solid arrow indicates the ripeness (completion of the ripening process). Different letters indicate the significant differences (*P* < 0.05) of *CHS. UFGT*, or *MybA1* expression among different treatments at each development stage.

The results from the anthocyanin measurements were matched by visual assessment of veraison (**Supplementary Figure S1**). The photographs in **Supplementary Figure S1** demonstrate that the application of ABA advanced coloring, and fluridone delayed coloring. Consequently, the onset of veraison of ‘Beihong’ berries was advanced by applying ABA at 20 and 10 days before veraison, while it was delayed by applying fluridone. The expression of genes related to anthocyanin biosynthesis was further investigated when exogenous ABA application or fluridone was conducted at 20 or 10 days pre-veraison. The expression levels of *CHS. UFGT*, and *MybA1* related to anthocyanin biosynthesis in the skin of ABA-treated berries were higher than in the control. Expression of the above three genes was reduced by application of the inhibitor when the treatment was conducted at 10 days pre-veraison, but was not changed when the treatment was conducted at 20 days pre-veraison (**Figure 5**).

### Effect of Exogenous ABA and Its Inhibitor Fluridone on Resveratrol Concentration in Berry Skin during Different Development Stages

**FIGURE 6:**
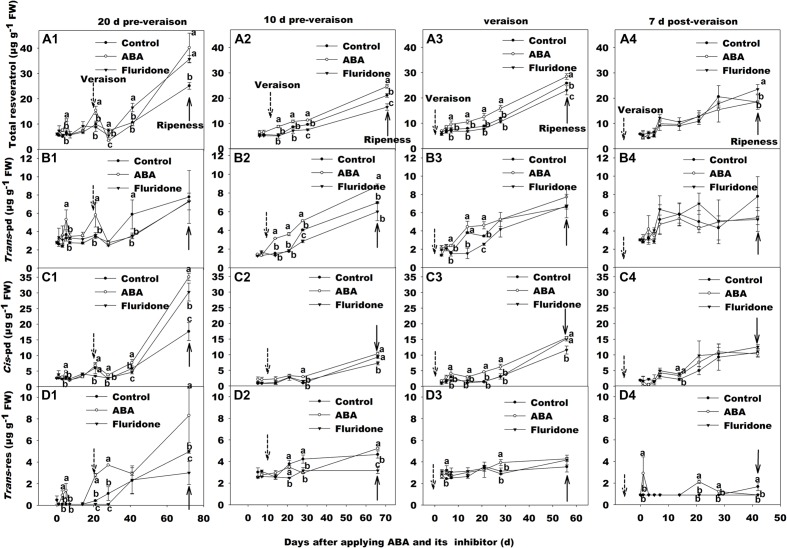
**Change of total resveratrol, *trans*-resveratrol (*trans*-Res), *trans*-piceid (*trans*-Pd), and *cis*-piceid (*cis*-Pd) concentration in berry skin of ‘Beihon*g*’ after applied with ABA and its synthetic inhibitor fluridone at 20 days pre-veraison** **(A1, B1, C1, and D1)**, 10 days pre-veraison **(A2, B2, C2, and D2)**, veraison **(A3, B3, C3, and D3)**, and 7 days post-veraison **(A4, B4, C4, and D4)**. Control, deionized water; ABA treatment, (+)-abscisic acid (500 mg L^-1^); Fluridone, an inhibitor of ABA synthesis (500 mg L^-1^). Dotted arrow indicates the beginning of veraison, solid arrow indicates the ripeness (completion of the ripening process). Different letters indicate the significant differences (*P* < 0.05) of total resveratrol, *trans*-Res, *trans*-Pd, or *cis*-Pd concentration among different treatments at each development stage.

When exogenous ABA or fluridone treatments were conducted at 20 days pre-veraison or 7 days post-veraison, there were no significant differences in resveratrol concentration between the treatments and the controls (**Figures 6A1–D1,A4–D4**). However, significant effects of both ABA and fluridone application were observed when the application occurred 10 days pre-veraison, and a significant effect of ABA application was observed when the treatment occurred at veraison (**Figures 6A2,A3**).

In the case of ABA application, the increase in resveratrol occurred approximately 10 days after application, whether applied 10 days pre-veraison or at veraison, the difference remaining stable until maturity, i.e., the rate of resveratrol accumulation after this point was almost the same in ABA-treated or control berries (**Figures 6A2,A3**). Furthermore, the increase in total resveratrol was mainly due to the two piceids (**Figures 6B2,B3,C2,C3**), while *trans*-res remained at a low level, not significantly different from the control (**Figures 6D2,D3**).

Total resveratrol concentration in the skins of berries treated with fluridone at 10 days pre-veraison was not affected by the treatment on the same timescale as the effect of ABA application. The difference appeared to be in the rate of accumulation post-veraison, being significant from 28 days after the treatment onward (**Figure 6A2**).

**FIGURE 7:**
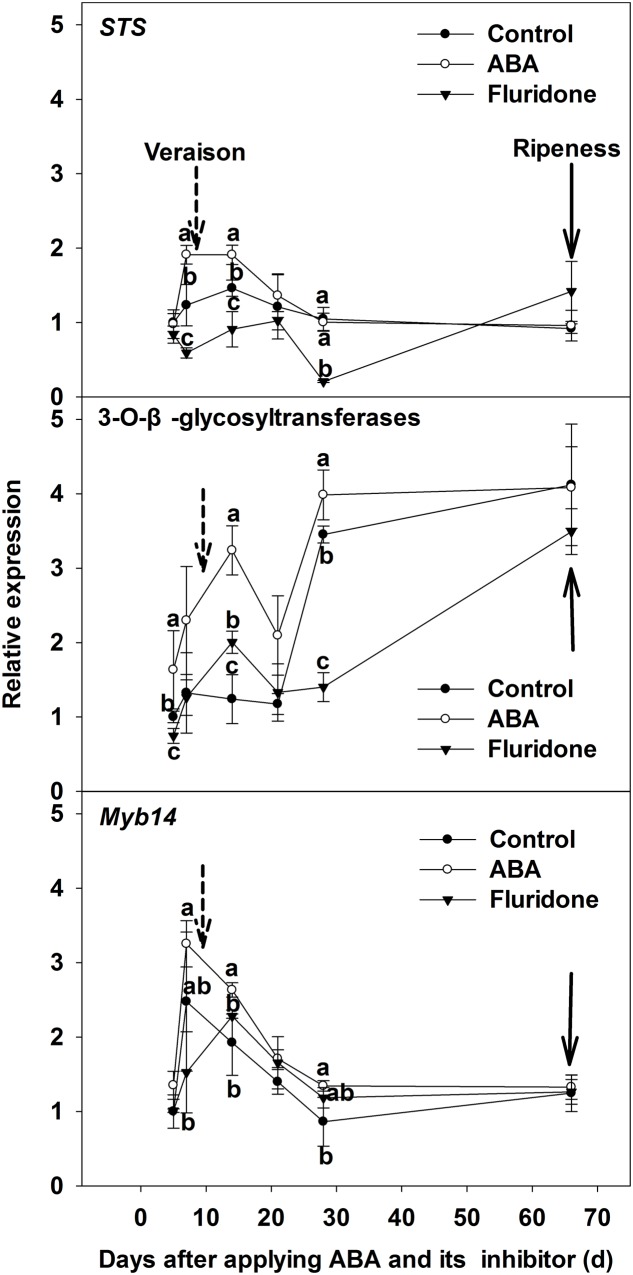
**Expression of genes *STS*, *3-O-GT* and *Myb14* related to resveratrol synthesis in berry skin of ‘Beihong’ after applied with ABA and its synthetic inhibitor fluridone at 10 days pre-veraison.** STS, stilbene synthase; 3-O-GT, 3-*O*-β-glycosyltransferases. Control, deionized water; ABA treatment, (+)-abscisic acid (500 mg L^-1^); Fluridone, an inhibitor of ABA synthesis (500 mg L^-1^). Dotted arrow indicates the beginning of veraison, solid arrow indicates the ripeness (completion of the ripening process). Different letters indicate the significant differences (*P* < 0.05) of *STS*, *3-O-GT* or *Myb14* expression among different treatments at each development stage.

We then investigated the expression of the genes related to resveratrol biosynthesis for the treated skins at 10 days pre-veraison (**Figure 7**). For the key gene of resveratrol biosynthesis, *STS*, the expression level in the ABA-treated berries reached a peak at 7 days after ABA application and was significantly higher than the control, then decreased to the level of the control at 21 days, remaining similar to controls until maturity. The expression level of *STS* in skins from ABA synthesis inhibitor treated berries was significantly lower than the control 7 days after application, but increased to the level of the control at 21 days (**Figure 7**). The expression of *Myb14*, an important regulatory gene of *STS*, was similar to *STS* following application of ABA or the inhibitor (**Figure 7**). The expression level of *3-O-GT* in the berry skin, the key gene related to the biosynthesis of two piceids, was higher than the control after applying ABA, but was lower than the control after applying the inhibitor (**Figure 7**).

## Discussion

As has been previously observed (Rogiers et al., 2004; Wheeler et al., 2009; Sun et al., 2010; Ferrara et al., 2015), there was an abrupt increase in berry ABA concentration in non-treated fruit immediately preceding the rapid rise in total anthocyanin concentration during veraison. Although ABA levels started to decrease 3 weeks post-veraison, anthocyanin concentration continued to rise throughout the ripening period, albeit at a much lower rate (**Figure 1B**). Wheeler et al. (2009) suggested that ABA triggers, but is not necessarily required to sustain, berry coloration. Our data, however, suggest that some ABA may be required for ongoing anthocyanin synthesis, as ABA remained at a higher level throughout maturation relative to the pre-veraison period (**Figures 1B,C**). Moreover, in the present study, exogenous ABA application accelerated coloring of the berry skin, i.e., advanced veraison, while the inhibitor of ABA synthesis (fluridone) delayed coloring, i.e., slowed veraison, when applied 20 and 10 days before veraison (**Supplementary Figure S1**). ABA is a hormone which plays a key role in fruit ripening (Finkelstein and Rock, 2002). However, to the best of our knowledge, a detailed comparison of ABA application at different development stages around veraison on endogenous ABA concentration in grape berry has not previously been undertaken. As expected, application of ABA strongly increased the level of the hormone in the skin, irrespective of developmental stage (**Figure 2**). The results indicated that a considerable amount of applied ABA should enter into the berry (∼0.5 μl of ABA solution per berry), or applying exogenous ABA stimulated a large increase in ABA biosynthesis and accumulation in the treated berries. The latter theory is supported by an increase in the transcript levels of *NCED2* and *NCED1* in ABA-treated berry skin compared with the controls 5, 7 days after the ABA application (**Figure 3**). Sun et al. (2010) and Ren et al. (2011) also had similar results. However, the details mechanism is worth further exploring.

At present, there has been limited evidence for a relationship between ABA and the biosynthesis of resveratrol. Ban et al. (2000) reported that exogenous ABA application at veraison increased resveratrol concentration by 20% in ripening ‘Kyoho’ grape berries, but no statistically significant difference was demonstrated. Deis et al. (2011) found that exogenous ABA can increase the concentration of resveratrol in grape berry skin in combination with water deficit. Recently, over-expression of the ABA responsive transcription factor *VvABF2* in grape cell suspension enhanced the response to ABA application, including the biosynthesis of resveratrol (Nicolas et al., 2014).

In the present study, resveratrol concentration gradually increased, from 3 weeks after veraison until maturity (**Figure 1**), the onset coinciding with the post-veraison peak in berry ABA concentration and, therefore, indicating a possible correlation between veraison and resveratrol biosynthesis in berry skin. In support of this, the application of exogenous ABA at 10 days pre-veraison both advanced veraison and increased the accumulation of resveratrol, whereas the inhibitor of ABA synthesis both postponed veraison and decreased the accumulation of resveratrol. These effects were maintained for 50–70 days (**Figures 2A2,A3**). In addition, the application of exogenous ABA or the inhibitor of ABA synthesis at veraison also increased or decreased the accumulation of resveratrol.

The accumulation of total resveratrol, following ABA application, was primarily in the form of the two piceids, which was also the case for the post-veraison accumulation of total resveratrol in the control (**Figures 6B2,B3,C2,C3**). ABA application also led to the increase of the expression of the genes related to the synthesis of resveratrol, *STS. 3-O-GT and Myb14*, while treatment with the ABA synthesis inhibitor resulted in a decrease in expression levels of these genes (**Figure 7**). In all treatments (control, ABA and fluridone), expression of *STS. 3-O-GT*, and *Myb14* increased or decreased around veraison, with a subsequent general increase in the expression level of *3-O-GT* toward maturity. These results confirm that resveratrol biosynthesis and accumulation is associated with veraison and modulated by ABA in berry skin.

The application of exogenous ABA at 20 days pre-veraison or 7 days post-veraison did not significantly affect the accumulation of resveratrol (**Figures 6A1,A4**), indicating that the sensitivity of resveratrol biosynthesis to ABA varies with developmental stage. Further, the contrast with anthocyanin accumulation, which occurred with ABA application at all developmental stages (**Figure 4**), demonstrates a difference in regulation between the two, despite sharing ABA responsiveness.

Holl et al. (2013) reported that *Myb14* specifically regulates resveratrol biosynthesis in grape. *MybA1* is also known to regulate the biosynthesis of anthocyanins (Jeong et al., 2006). The 5′-flanking sequence (1.6 kb) of *MybA1* was found to contain ABRE binding *cis*-elements, which were also found in cherry skin, when analyzed using the PLACE website (Shen et al., 2014). In our study, motif analysis of 2 kb region upstream of start codon, also revealed ABRE binding *cis*-elements for *Myb14* (**Supplementary Table S2**). Interestingly, our study found that exogenous ABA application led to an increase of the expression level of both *Myb14 and MybA1*, while the inhibitor application resulted in a decrease in their expression (**Figure 7**). These results suggest that ARBE/ABFs could be involved in ABA-induced resveratrol and anthocyanin biosynthesis. Nicolas et al. (2014) reported that *VvABF2* over-expression stimulated a range of ABA responsive genes, as well as resveratrol production in a grape cell suspension. Consequently, *VvABF2* maybe have a role in ABA-induced resveratrol and anthocyanin synthesis through the expression of *Myb14* and *MybA1*, although the signal transduction pathway from ABA to *Myb14* needs further research.

## Conclusion

Our results suggest the synthesis and accumulation of resveratrol during development of ‘Beihong’ (*V. vinifera* × *V. amurensis*) berries was directly related to veraison. Further, that this accumulation, together with that of anthocyanins, was associated with berry ABA concentration. The response of resveratrol biosynthesis in the berry skin to exogenous ABA varied with development stage, being responsive only when close to veraison and was less sensitive to ABA than anthocyanin biosynthesis.

## Author Contributions

LW and SL conceived the study. JW and SW performed the experiments and conducted statistical analysis. LW and JW wrote the manuscript. GL and WD helped to carry out primary and secondary metabolite detection. SL and EE conducted ABA determination and revised the manuscript. All the authors read and approved the final manuscript.

## Conflict of Interest Statement

The authors declare that the research was conducted in the absence of any commercial or financial relationships that could be construed as a potential conflict of interest.

## Supplementary Material

**FIGURE S1 | Photographs of ‘Beihong’ berries applied with ABA and its synthetic inhibitor fluridone at 10 days pre-veraison.** Control, deionized water; ABA treatment, (+)-abscisic acid (500 mg L^-1^); Fluridone: an inhibitor of ABA synthesis (500 mg L^-1^). The photographs from top to below indicate 5, 7, 14, 21, 28, and 66 days after applications.

**TABLE S1 | Primers of the genes related to resveratrol, anthocyanin and ABA synthesis.**

**TABLE S2 |*Cis*-acting elements potentially associated with the *Myb14* in grape berry skin.** TIS, translation initiation site.

## Abbreviations

ABA: abscisic acid
DAA: days after anthesis
HPLC: high performance liquid chromatography
LC-MS/MS: liquid chromatography/mass spectrometry
