# Supplementary material for: The Synthesis and Accumulation of Resveratrol Are Associated with Veraison and Abscisic Acid Concentration in Beihong (Vitis vinifera × Vitis amurensis) Berry Skin
Source: Front Plant Sci. 2016 Nov 3;7:1605. doi: 10.3389/fpls.2016.01605 (PMC5094005; doi:10.3389/fpls.2016.01605)
Supplement: Supplementary file 1 [file Image_1.PDF]

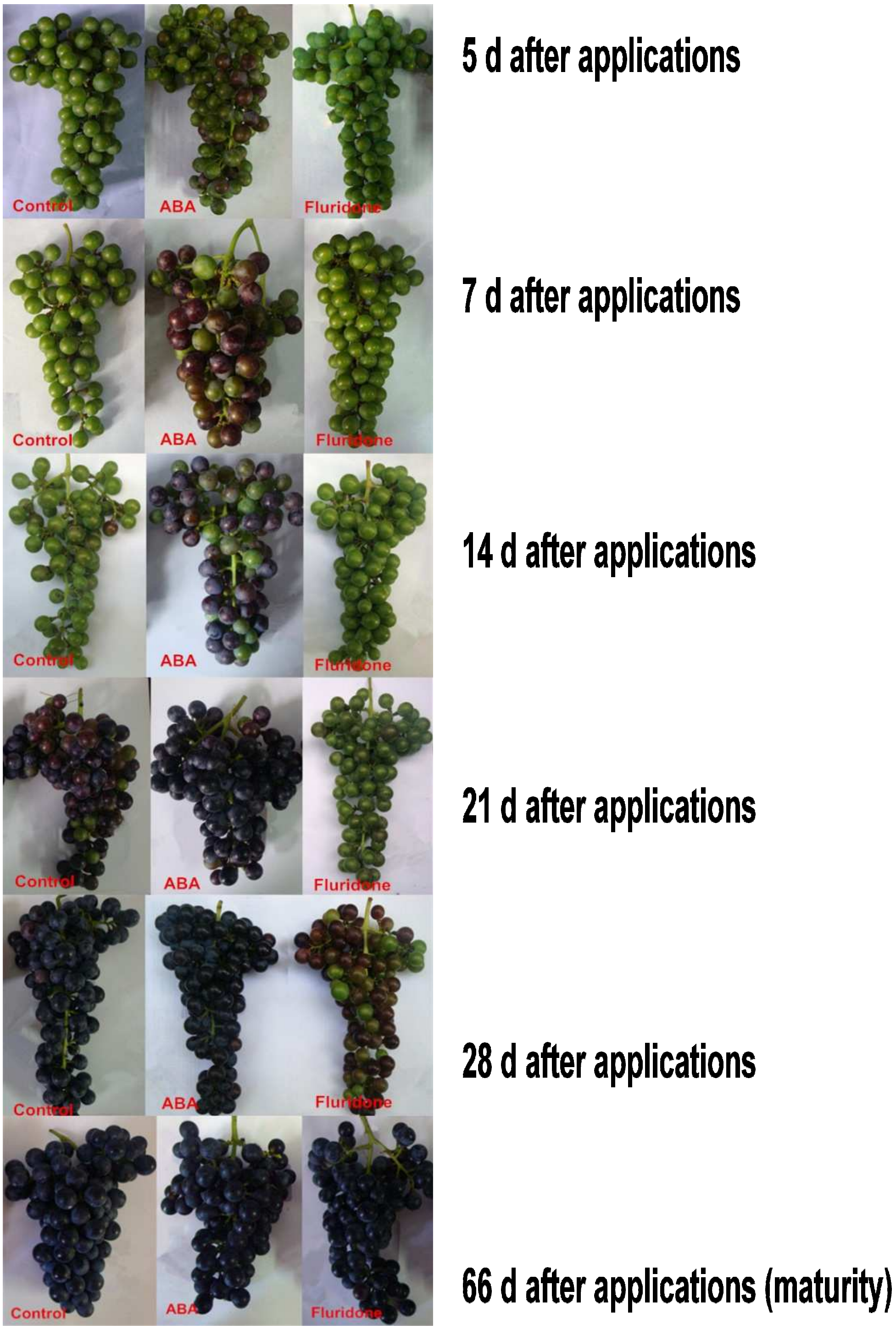

Supplementary image
