# Supplementary material for: The Synthesis and Accumulation of Resveratrol Are Associated with Veraison and Abscisic Acid Concentration in Beihong (Vitis vinifera × Vitis amurensis) Berry Skin
Source: Front Plant Sci. 2016 Nov 3;7:1605. doi: 10.3389/fpls.2016.01605 (PMC5094005; doi:10.3389/fpls.2016.01605)
Supplement: Supplementary file 2 [file Table_1.PDF]

Supplementary table 1

Gene name	Accession no.		Primers
Actin	VIT_04s0044g00580	**Forward:**	**CTTGCATCCCTCAGCACCTT**
		**Reverse:**	**TCCTGTGGACAATGGATGGA**
STS	VIT_16s0100g00750[a]	**Forward:**	**TTAGAAACGCTCAACGTGCCAAGGG**
		**Reverse:**	**AATCAGCATAATCAGACTGGTAGAC**
O-3-GT	VIT_03s0180g00200[b]	**Forward:**	**GGCTTCAAAGGGCTTGCTTGTG**
		**Reverse:**	**TCTGAGGCCGGATATCAAAC**
Myb14	VIT_07s0005g03340	**Forward:**	**GGGACGCATCAAGAGAGTGT**
		**Reverse:**	**GTGCTGTCATTGGCAGTAG**
CHS	VIT_14s0068g00920	**Forward:**	**GAAGATGGGAATGGCTGCTG**
		**Reverse:**	**AAGGCACAGGGACACAAAAAG**
UFGT	VIT_16s0039g02230	**Forward:**	**GGGATGGTAATGGCTGTGG**
		**Reverse:**	**ACATGGGTGGAGAGTGAGTT**
MybA1	VIT_02s0033g00410	**Forward:**	**TAGTCACCACTTCAAAAAGG**
		**Reverse:**	**GAATGTGTTTGGGGTTTATC**
NCED1	VIT_19s0093g00550	**Forward:**	**GAGACCCCAACTCTGGCAGG**
		**Reverse:**	**AAGGTGCCGTGGAATCCATAG**
NCED2	VIT_10s0003g03750	**Forward:**	**AGTTCCATACGGGTTTCATGGG**
		**Reverse:**	**CCATTTTCCAAATCCAGGGTGT**

[a] Besides *VvSTS7* (VIT_16s0100g00750), the detected *STSs* also include 12 *STS* genes which are *VvSTS9* (VIT_16s0100g00770), *VvSTS10* (VIT_16s0100g00780), *VvSTS15* (VIT_16s0100g00830), *VvSTS21* (VIT_16s0100g00910), *VvSTS27* (VIT_16s0100g00990), *VvSTS29* (VIT_16s0100g01010), *VvSTS33* (VIT_16s0100g01060), *VvSTS37* (VIT_16s0100g01110), *VvSTS41* (VIT_16s0100g01130), *VvSTS45* (VIT_16s0100g01160), *VvSTS47* (VIT_16s0100g01190) and *VvSTS48* (VIT_16s0100g01200). The specific primers were designed according to their common sequences.

[b] The detected *O-3-GTs* included two members which are *VvO-3-GT1* (VIT_03s0180g00200) and *VvO-3-GT2* (VIT_03s0180g00320). The specific primers were designed according to their common sequences.
