# Supplementary material for: The Synthesis and Accumulation of Resveratrol Are Associated with Veraison and Abscisic Acid Concentration in Beihong (Vitis vinifera × Vitis amurensis) Berry Skin
Source: Front Plant Sci. 2016 Nov 3;7:1605. doi: 10.3389/fpls.2016.01605 (PMC5094005; doi:10.3389/fpls.2016.01605)
Supplement: Supplementary file 3 [file Table_2.PDF]

Supplementary table 2

Gene	Strand	Distance from TIS	Sequence
Myb14	+	**823**	**YACGTGGC**
	+	**822**	**RYACGTGGYR**
	+	**416**	**ACGTG**
	+	**824**	**ACGTG**
	**-**	**323**	**ACGTG**
	**-**	**415**	**ACGTG**
	**-**	**739**	**ACGTG**
	+	**415**	**MACGYGB**
	**-**	**414**	**MACGYGB**
	**-**	**738**	**MACGYGB**
	+	**1661**	**ACGTG**
	+	**1747**	**ACGTG**
	**-**	**1746**	**ACGTG**
	+	**1746**	**MACGYGB**
	**-**	**1745**	**MACGYGB**
